# Age of Migration and the Health Status of Older Latinos: Findings From the Health and Retirement Study

**DOI:** 10.1093/geroni/igab046.1752

**Published:** 2021-12-17

**Authors:** Blakelee Kemp, Marc Garcia

**Affiliations:** 1 University of Nebraska, Nebraska, United States; 2 Syracuse University, Syracuse, New York, United States

## Abstract

Life course research emphasizes the importance of considering how early life experiences set individuals on specific trajectories over time with implications across multiple health domains. Life experiences of older Latinos are shaped by where they were born and, for the foreign-born, when they immigrated to the United States. Prior research examining the extent to which age of migration is associated with health has largely been limited to regional studies. To address this gap in knowledge, we use nationally representative data from the Health and Retirement Study to examine associations between age of migration and multiple physical health outcomes among older Latinos residing in the United States. We examine 2010 prevalence and follow-up incidence to 2016 of cardiovascular issues, diabetes, one or more activities of daily living (ADLs), one or more instrumental activities of daily living (IADLs), cognitive issues, and mortality incidence. Preliminary results indicate similar health profiles across Latinos who migrated in early life (<18), during adulthood (18-34), and during later adulthood (35+). Most health profiles were similar among Latino men and women except for prevalence and incidence of experiencing difficulties with at least one ADL. Latino women who migrated in later-adulthood have higher prevalence of ADLs and women who migrated early in life (>18) have higher ADL incidence than Latino men who migrated during the same life course periods. A greater understanding of the how immigrant experiences influence physical health outcomes offers important insights into the development of actionable and culturally appropriate social and health policies.

